# Telemedicine to Improve Medical Care of Fishermen in Pelagic Fisheries

**DOI:** 10.3390/healthcare14010058

**Published:** 2025-12-25

**Authors:** Po-Heng Lin, Chih-Che Lin

**Affiliations:** 1 Division of General Surgery, Department of Surgery, Kaohsiung Chang Gung Memorial Hospital, Chang Gung University College of Medicine, Kaohsiung 83301, Taiwan; 2 Kaohsiung Municipal Fong Shan Hospital—Under the Management of Chang Gung Medical Foundation, Kaohsiung 83091, Taiwan

**Keywords:** telemedicine, pelagic fisheries, maritime healthcare, satellite communication, artificial intelligence, wearable health monitoring

## Abstract

Fishermen operating in pelagic fisheries often experience significant barriers to medical care due to geographic isolation, harsh environmental conditions, and the absence of onboard healthcare personnel. Telemedicine offers an effective approach to overcome these limitations by enabling remote diagnosis, monitoring, and treatment through satellite-based communication systems. This review summarizes the progress and applications of telemedicine in maritime and other austere environments, focusing on technological advancements, clinical implementations, and emerging trends in artificial intelligence-driven healthcare. Evidence from pilot and retrospective studies highlights the growing use of wearable devices, telementored ultrasound, digital photography, and cloud-based monitoring systems for managing acute and chronic medical conditions at sea. The integration of machine learning and deep learning algorithms has further improved fatigue, stress, and motion detection, enhancing early risk assessment among seafarers. Despite challenges such as limited connectivity, data privacy concerns, and training requirements, the adoption of telemedicine significantly improves health outcomes, reduces emergency evacuations, and promotes occupational safety. Future directions emphasize the development of 5G-enabled Internet of Medical Things networks and predictive AI tools to establish comprehensive maritime telehealth ecosystems for fishermen in pelagic operations.

## 1. Introduction

Fishermen engaged in pelagic fisheries operate in some of the most isolated and challenging environments on Earth, spending extended periods at sea where access to timely medical care is severely limited [1,2]. The combination of physical labor, unpredictable weather, long working hours, and limited onboard medical infrastructure exposes them to a wide range of occupational health risks, including injuries, dehydration, fatigue, and psychological stress. In most cases, vessels lack qualified medical personnel, and crew members rely on basic first-aid knowledge, which is often insufficient during medical emergencies. These constraints highlight a persistent gap in healthcare accessibility for maritime workers—one that modern telemedicine is uniquely positioned to fill. Telemedicine, defined as the remote delivery of clinical healthcare using telecommunications and digital technology, has emerged as a practical and transformative approach to maritime healthcare [3]. By integrating satellite communication, portable diagnostic devices, and cloud-based data platforms, telemedicine enables real-time consultation between onboard crews and onshore medical professionals. This digital link allows for immediate evaluation of injuries, medical events, or psychological distress—conditions that previously required long delays or emergency evacuations. The evolution from traditional radio-based medical advice to interactive, video-enabled teleconsultation marks a major shift in how healthcare is delivered at sea, turning distant ships into mobile healthcare units connected to expert medical networks [4].

Recent technological advancements have accelerated this transition. Wearable biosensors now enable continuous monitoring of vital parameters such as heart rate, blood oxygen levels, and stress indicators, providing early warning of physiological deterioration. Portable ultrasound and electrocardiogram (ECG) devices allow for onboard imaging and cardiac assessments that can be transmitted instantly to telemedical assistance centers for interpretation. Meanwhile, the integration of cloud computing and artificial intelligence (AI) has enhanced data management and diagnostic precision, allowing for predictive health analysis and early intervention. These innovations together create a connected healthcare ecosystem capable of supporting both acute emergency response and long-term preventive health surveillance. The maritime sector’s adoption of telemedicine extends beyond immediate medical care; it represents a broader commitment to occupational safety, crew welfare, and sustainable fisheries management. For fishermen in pelagic operations, where voyages can span weeks or months, the ability to receive continuous medical supervision and expert consultation fundamentally improves health outcomes, operational efficiency, and morale. Moreover, the lessons learned from telemedicine in other extreme environments, such as Antarctic research bases and mountain expeditions, reinforce its reliability under austere conditions [4,5,6].

Hat et al. introduced the concept of the Human Digital Twin (HDT) as a digital replica of an individual designed to integrate human characteristics directly into system modeling and operational decision-making. This framework aims to revolutionize human–system interaction in maritime environments by enhancing situational awareness, predicting crew performance, and improving overall system reliability. However, the current development of HDT models has given limited attention to the cognitive processes that govern human perception, judgment, and decision-making during complex maritime operations. To address this gap, Hat et al. proposed a model-based framework that integrates the Information–Decision–Action of Crew model with a Discrete Dynamic Event Tree to dynamically capture changes in human cognition and behavior under varying conditions [7]. Their case study, conducted using a ship hydrodynamic simulator, demonstrated that the Cognitive Process-based HDT can quantitatively represent fluctuations in crew states and response mechanisms, offering a promising pathway for future telemedicine and maritime safety systems that rely on real-time human–machine integration.

Previous reviews have examined telemedicine applications in maritime shipping, polar expeditions, offshore energy platforms, and space-analog environments such as NASA’s NEEMO (NASA Extreme Environment Mission Operations) and HI-SEAS (Hawaii Space Exploration Analog and Simulation) missions. These studies typically focus on structured, institution-supported contexts with stable communication links, designated medical officers, and standardized emergency-response protocols. Similarly, reviews of Arctic and Antarctic expedition medicine emphasize telehealth integration within well-equipped research stations characterized by scheduled operations and robust logistical support. In contrast, fishermen engaged in pelagic operations experience far more variable and resource-limited conditions. Fishing vessels often lack trained medical personnel, operate with unpredictable travel routes, and rely on intermittent satellite connectivity while facing high rates of injuries, fatigue, environmental exposure, and delayed evacuation. Despite constituting one of the world’s most hazardous occupations, this group remains underrepresented in broader telemedicine syntheses. This review specifically addresses that gap by integrating technological, clinical, and AI-enabled perspectives tailored to the unique constraints of pelagic fisheries, highlighting operational realities that differ substantially from other austere telemedicine models. Finally, it discusses the benefits, challenges, and future perspectives of maritime telehealth, emphasizing the need for international collaboration to establish scalable and standardized systems capable of safeguarding the health and well-being of seafarers worldwide.

## 2. Technological Framework for Maritime Telemedicine

The advancement of telemedicine in maritime environments represents a transformative milestone in the way medical care is provided to individuals working in remote, high-risk occupations such as pelagic fisheries. Unlike terrestrial healthcare systems, where patients and clinicians can interact face-to-face or through stable network infrastructures, maritime healthcare delivery depends on the seamless coordination of satellite communication, onboard diagnostic technologies, data management systems, and artificial intelligence–driven analytical tools. The entire ecosystem must operate reliably under the constraints of unpredictable weather, limited power supply, and scarce medical personnel. As a result, the technological framework for maritime telemedicine must emphasize not only real-time connectivity but also system resilience, interoperability, and data security to ensure continuous and accurate healthcare support for fishermen operating far from the shore.

### 2.1. Ship-to-Shore Communication Systems

**Figure 1:**
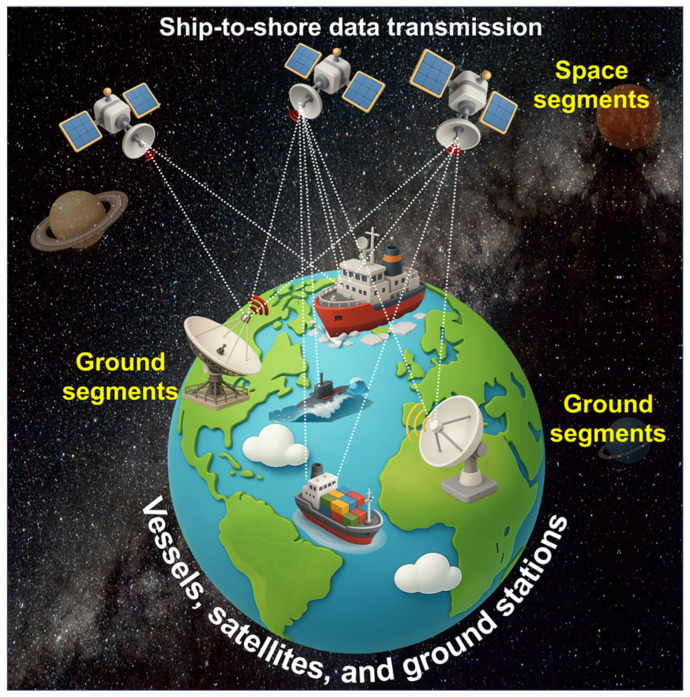
Ship-to-shore data transmission through satellite.

At the core of maritime telemedicine lies the ship-to-shore communication infrastructure, which enables the continuous transmission of medical information between vessels and onshore healthcare centers. This system is primarily powered by satellite networks such as Inmarsat FleetBroadband, Iridium Certus, and Very Small Aperture Terminal (VSAT) systems, which provide broadband connectivity across global maritime zones. Through these satellite links, clinical data, including electrocardiograms, ultrasound images, and real-time video streams, can be transmitted to telemedical assistance services (TMAS) that operate around the clock. Figure 1 illustrates the operational flow of this communication chain, where data originating from the ship’s medical workstation or wearable health devices are transmitted to orbiting satellites and relayed to receiving ground stations. These data are subsequently routed to hospitals, universities, or maritime telehealth centers, where physicians interpret the information and provide immediate diagnostic or therapeutic guidance [8,9].

This communication model enables both synchronous and asynchronous telemedicine. In the synchronous mode, live video conferencing allows physicians to interact directly with the crew, observe symptoms, and guide emergency medical interventions such as wound suturing, defibrillation, or administration of intravenous therapy. In asynchronous communication, or “store-and-forward” telemedicine, medical data such as dermatological photographs or ultrasound scans are stored locally and transmitted when the connection stabilizes, ensuring continuous care despite bandwidth interruptions. The integration of these communication modalities has drastically reduced response times for medical emergencies at sea, minimized unnecessary helicopter evacuations, and increased diagnostic accuracy for conditions like cardiac arrhythmia, trauma, and infectious diseases. This framework is particularly vital for pelagic fishermen, who often remain at sea for weeks without access to land-based healthcare facilities, relying entirely on ship-to-shore connectivity for medical intervention [10].

### 2.2. Cloud-Based Monitoring and Data Integration

**Figure 2:**
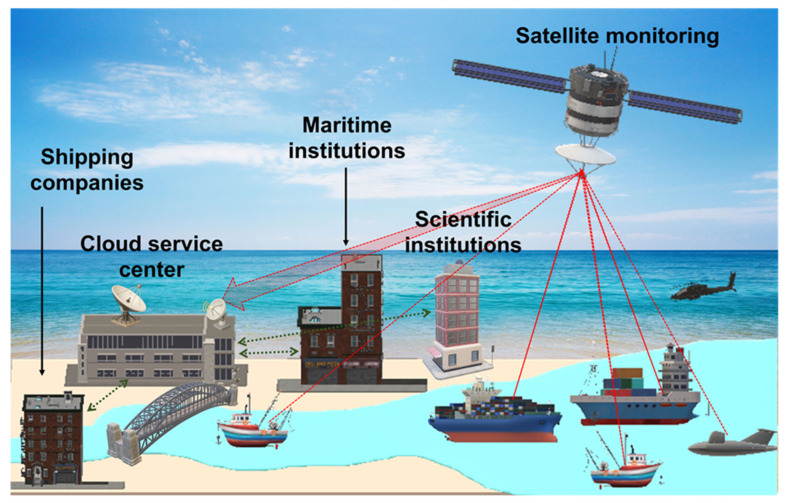
Practical applications for ship-to-shore data transmission by satellite monitoring through a cloud service center to shipping companies, maritime institutions, and scientific institutions.

The second pillar of the maritime telemedicine framework is the incorporation of cloud computing for data management and real-time monitoring. Figure 2 represents this architecture, wherein medical data collected aboard vessels is transmitted through encrypted satellite links and stored within centralized cloud servers. These cloud systems serve as digital repositories that connect shipping companies, telemedical centers, maritime regulatory bodies, and scientific institutions under a unified data-sharing network. The cloud infrastructure ensures that each seafarer’s medical record, including diagnostic images, consultation histories, and physiological parameters, remains accessible to authorized healthcare professionals regardless of location. By integrating various data streams, this model creates a continuous feedback loop between ship and shore, fostering collaborative decision-making during emergencies and improving preventive healthcare planning [11].

Cloud-based systems also enable predictive analytics by processing large datasets accumulated from wearable devices, environmental sensors, and medical instruments. Artificial intelligence algorithms analyze fluctuations in physiological parameters, such as heart rate variability, body temperature, and oxygen saturation, to predict health risks like fatigue, heat stress, or dehydration before symptoms become clinically evident. Additionally, the combination of environmental monitoring data (including sea temperature, humidity, and air pressure) with physiological parameters allows for predictive modeling of occupational hazards specific to pelagic operations. During the COVID-19 pandemic, cloud-integrated telemedicine networks proved indispensable in managing onboard disease surveillance and quarantine coordination. Data integration ensured immediate triage and isolation protocols for suspected cases, thereby preventing onboard transmission and enabling safe continuation of fishing operations. Consequently, cloud-based telemedicine has evolved from a passive data storage model into an active, decision-supporting infrastructure for global maritime healthcare [12].

### 2.3. Integration of Portable Diagnostic Devices

The development and miniaturization of medical diagnostic instruments have revolutionized the feasibility of telemedicine aboard fishing vessels. Modern maritime telehealth systems now incorporate compact, ruggedized devices capable of providing hospital-grade diagnostics in confined shipboard environments. Handheld ultrasound scanners, for example, allow operators to capture high-quality images of internal organs, musculoskeletal structures, or cardiac conditions, which can be transmitted instantly via satellite to shore-based specialists. This innovation has proven particularly effective for diagnosing internal injuries, pneumothorax, or pericardial effusions during voyages when immediate evacuation is not possible. Similarly, portable ECG units and automated external defibrillators (AEDs) enable early detection and response to cardiovascular events—a leading cause of morbidity among fishermen and seafarers. Wearable health monitoring devices, such as wristbands or chest straps equipped with biosensors, continuously record heart rate, oxygen saturation, movement, and sleep patterns, transmitting these data in real time to cloud servers for medical supervision [5,13].

The adoption of these portable devices has not only improved emergency preparedness but also introduced a new paradigm of preventive healthcare at sea. Instead of focusing solely on crisis intervention, fishermen can now benefit from continuous physiological monitoring that helps detect fatigue accumulation, sleep disturbances, or dehydration before they impair performance. Studies summarized in this review confirm the growing reliability of these technologies in extreme environments, including polar expeditions and jungle operations, further validating their applicability in pelagic fisheries. By empowering crew members to perform first-line diagnostics under remote supervision, portable devices effectively extend the reach of medical expertise from hospitals to the open ocean, transforming vessels into floating health monitoring stations.

### 2.4. Artificial Intelligence and Decision Support Systems

The integration of AI represents a pivotal advancement in maritime telemedicine. Machine learning and deep learning algorithms can process large volumes of physiological and environmental data, enabling automated detection of abnormalities and generation of clinical alerts without requiring constant human oversight. As summarized, AI models such as Decision Tree, Random Forest, k-Nearest Neighbor, and Support Vector Machine have been applied for recognizing patterns of fatigue, stress, and motion among workers in demanding environments. More sophisticated deep learning models, including Convolutional Neural Networks (CNNs) and Bidirectional Long Short-Term Memory (BiLSTM) networks, have achieved remarkable accuracy in classifying mental fatigue and physical stress states, often exceeding 90% reliability. These analytical frameworks, when integrated with wearable devices aboard ships, can alert telemedical centers in real time when abnormal patterns are detected, such as elevated heart rate, reduced motion variability, or prolonged stress indicators [14].

AI-based decision support systems also facilitate predictive diagnostics and adaptive feedback. By continuously learning from historical health data and operational conditions, algorithms can anticipate the likelihood of specific medical events, such as heatstroke or cardiac arrhythmia, and automatically suggest preventive measures to crew members. The resulting system functions as a digital co-pilot for health, supplementing human medical judgment with algorithmic intelligence. For fishermen in pelagic operations, this predictive capability is particularly valuable because it allows for early intervention in environments where immediate evacuation is impractical. In essence, AI transforms telemedicine from a reactive support system into a proactive and anticipatory healthcare platform, aligning with the modern vision of precision maritime medicine [15].

### 2.5. Security, Interoperability, and System Resilience

The sensitive nature of medical data transmitted over global networks necessitates rigorous cybersecurity and interoperability standards. Maritime telemedicine systems employ multi-layer encryption, secure authentication protocols, and anonymization measures to safeguard patient information during transmission and storage. Given the diverse array of technologies involved, ranging from satellite and 4G/5G maritime broadband to shipboard Wi-Fi, interoperability becomes essential to ensure seamless communication between devices and networks. Systems must be designed to automatically switch between available communication channels based on signal strength and bandwidth availability, ensuring reliability even during adverse weather conditions or signal disruptions. Future advancements, including 5G-enabled maritime networks and the emerging Internet of Medical Things (IoMT), are expected to enhance bandwidth capacity and reduce latency, allowing for high-definition teleconsultations, real-time AI analytics, and continuous video diagnostics to become routine components of onboard healthcare [16,17].

Resilience is equally critical for the long-term sustainability of telemedicine at sea. Equipment must withstand vibration, humidity, and saline corrosion, while software systems should be capable of autonomous operation when temporarily disconnected from the cloud. Training programs for crew members are vital to ensure the correct usage of medical devices and adherence to teleconsultation protocols. The resilience of these systems thus depends not only on technology but also on human readiness and institutional collaboration among maritime operators, telehealth providers, and regulatory agencies. Together, these components create an adaptive ecosystem capable of maintaining healthcare delivery under the unpredictable conditions of pelagic fisheries.

## 3. Review of Telemedicine Studies in Maritime and Austere Environments

This provides a structured overview of the breadth of telemedicine research conducted across maritime and other isolated environments, categorizing the works by type of publication, environmental context, and thematic focus. The distribution of studies reveals a predominant emphasis on maritime healthcare, reflecting the global priority of improving medical accessibility for seafarers and fishermen working under physically demanding and geographically isolated conditions. Compared to other remote settings such as Antarctic stations, mountainous expeditions, or jungle operations, the maritime environment exhibits the highest concentration of telemedicine activities, underscoring its importance as a model setting for testing remote healthcare systems.

The entries in Table 1 are organized according to article type—pilot studies, case reports, qualitative studies, company reports, retrospective analyses, and commentaries—demonstrating a progressive evolution from feasibility assessments to structured telemedical operations. Pilot studies occupy a significant portion of the table, primarily addressing the initial deployment and performance evaluation of telemedical devices and systems. These include wearable digital health monitors, portable ultrasound imaging units, and telemonitoring tools for real-time physiological tracking. The recurring inclusion of ultrasound and telementoring categories indicates the central role of imaging and guided diagnostics in establishing reliable remote healthcare for maritime and expeditionary settings. The feasibility of ultrasound-based diagnosis and tele-guided procedures through satellite communication highlights the growing technological maturity of telemedicine in environments where conventional healthcare is unavailable.

## 4. Medical Case Studies and Observations in Maritime Healthcare

The data compiled in Table 2 provide a comprehensive overview of how telemedicine has been applied across maritime operations through retrospective, observational, descriptive, and epidemiological analyses. Collectively, these studies highlight the evolution of telemedicine from isolated case-based support to a structured healthcare system capable of both emergency response and preventive medical management for seafarers. The findings encompass a broad range of sample sizes, from small clinical groups to extensive datasets exceeding several thousand maritime health cases, reflecting the increasing institutionalization of telemedical assistance at sea. Retrospective investigations demonstrate that telemedicine significantly improves the efficiency of medical response aboard ships. By enabling continuous ship-to-shore communication, medical assistance services can remotely assess the severity of onboard health incidents, provide immediate clinical guidance, and determine whether evacuation is necessary. This model has been instrumental in reducing the frequency of unnecessary emergency transfers, optimizing logistics, and minimizing operational downtime. The capacity to manage most injuries, infections, and acute illnesses remotely illustrates that teleconsultation has become a cornerstone of maritime health safety. Moreover, the recorded cases show that long-term implementation of telemedical systems enhances the consistency and documentation of health incidents, supporting occupational health surveillance across fleets.

Observational and descriptive analyses reinforce the role of telemedicine in broadening the scope of maritime healthcare. The use of teleconsultation platforms and remote diagnostic tools has enabled efficient management of both acute and chronic conditions, including cardiovascular disorders, musculoskeletal injuries, and dermatological issues. Crew members, often with limited medical training, can now perform guided diagnostic procedures such as ECG recording, basic wound care, and ultrasound imaging under real-time supervision from onshore specialists. This capability allows for prompt diagnosis, stabilization, and targeted treatment without delay. Chronic disease management has also benefited substantially, as periodic health monitoring and digital medical records facilitate early detection of conditions that could otherwise progress unnoticed during prolonged voyages. Epidemiological findings presented in the dataset reveal recurring patterns in maritime occupational health, such as higher injury and illness rates among specific job categories and age groups. Telemedicine has proven effective in identifying these trends through systematic data collection, contributing to the design of preventive programs and policy interventions aimed at improving crew welfare. The integration of continuous telemonitoring and digital reporting has allowed shipping companies and health authorities to analyze health statistics in real time, translating clinical data into actionable safety measures. This capacity for large-scale data aggregation positions telemedicine not only as a clinical service but also as an essential component of maritime public health surveillance.

A particularly important aspect of these findings concerns the application of telemedicine during the COVID-19 pandemic, which served as a global validation of its value in emergency preparedness and outbreak control. Remote consultation systems were rapidly adapted to monitor symptoms, implement quarantine measures, and maintain communication between vessels and health authorities. Medical teams provided guidance for isolation, temperature tracking, and respiratory assessment, ensuring continuous operational safety while preventing disease transmission onboard. The pandemic also expanded the use of telemedicine for psychological support, addressing mental health challenges related to isolation and prolonged confinement. This period demonstrated that telemedicine can function as an integrated medical infrastructure, capable of supporting both clinical management and crisis mitigation under unprecedented conditions [3].

## 5. Integration of Artificial Intelligence in Telemedicine

The application of AI in telemedicine represents a major technological advancement that transforms traditional remote healthcare into an intelligent, adaptive, and predictive system. The summary presented in Table 3 highlights the growing implementation of AI models for health monitoring, focusing particularly on stress, fatigue, and motion detection. These models utilize various machine learning and deep learning architectures—including Decision Tree (DT), Random Forest (RF), k-nearest Neighbor (k-NN), Naive Bayes (NB), Support Vector Machine (SVM), CNN, Long Short-Term Memory (LSTM), and hybrid ensembles such as RF + SVM or CNN + LSTM + BiLSTM—to analyze physiological and behavioral data obtained from wearable or environmental sensors. Machine learning algorithms form the foundation of AI-based health monitoring by identifying subtle patterns in complex physiological datasets. In this framework, models such as DT, RF, and SVM are trained to classify variations in biometric signals—heart rate, body movement, or electroencephalogram (EEG) activity—that correspond to stress or fatigue levels. Their performance, as reflected in Table 3, shows reliability values between 70% and 85%, demonstrating their suitability for continuous physiological assessment in real-time maritime operations. Deep learning approaches extend this capability by employing multilayered neural networks that can automatically extract higher-order features from raw data. For example, CNN and BiLSTM architectures achieve accuracy levels exceeding 88% and, in some cases, approaching 99.9%, particularly when applied to mental fatigue classification using EEG inputs. Hybrid models that combine multiple algorithms provide even greater robustness, often surpassing 98% reliability, indicating strong potential for use in demanding and noisy environments like offshore fishing vessels. These AI systems are directly relevant to the health monitoring of fishermen who endure prolonged exposure to physically and mentally taxing marine conditions. Extended shifts, fluctuating temperatures, limited rest, and mechanical vibration collectively induce fatigue and stress—key factors influencing safety and productivity [57]. Integrating AI with wearable technologies allows for continuous assessment of these physiological responses, enabling early intervention before fatigue impairs decision-making or motor coordination. For example, an AI-enabled sensor network could alert ship officers when cumulative stress indices exceed safe thresholds, prompting mandatory rest periods or workload redistribution. The combination of algorithmic intelligence and sensor-based data acquisition transforms reactive medical assistance into a proactive safety management system, aligning telemedicine with preventive occupational health objectives. The reliability metrics summarized in Table 3 underscore the maturity of AI-assisted telemonitoring for real-world deployment. Consistently high accuracy levels across machine learning, deep learning, and hybrid categories suggest that these methods can effectively interpret data from non-invasive sensors such as smart bands, EEG headsets, or motion trackers. When integrated into maritime telemedicine frameworks, these AI models can operate through ship-to-shore cloud networks, allowing for real-time analysis and automated transmission of alerts to telemedical centers. Such integration not only enhances diagnostic precision but also reduces the cognitive and procedural workload on crew members, who can rely on automated systems to identify early signs of physiological decline [58]. In pelagic fisheries, most evidence supporting AI-assisted telemedicine originates from related occupational, clinical, or other austere environments rather than from large-scale validation studies conducted directly aboard fishing vessels. Although these studies indicate strong potential for AI-based fatigue, stress, and health monitoring among fishermen, direct validation under real pelagic fishing conditions remains limited. Accordingly, the AI applications discussed should be regarded as translational and prospective rather than fully validated for routine deployment.

## 6. Benefits and Challenges

### 6.1. Benefits

The implementation of telemedicine in maritime healthcare offers a range of tangible and far-reaching benefits, particularly for fishermen working in pelagic fisheries where conventional access to medical services is virtually impossible. One of the most significant advantages is the improved access to medical expertise. Through satellite-based communication and digital diagnostic tools, crew members can obtain direct consultation from qualified physicians and specialists located onshore. This eliminates the constraints of isolation and enables immediate professional evaluation of injuries, illnesses, or other medical emergencies. The ability to connect vessels to telemedical assistance centers ensures that expert advice is available around the clock, regardless of geographic location or environmental conditions. Another major benefit is the substantial reduction in response time and the associated cost of emergency evacuations. Before telemedicine was widely adopted, medical incidents at sea often required helicopter rescues or emergency docking—operations that were both logistically complex and financially burdensome. Telemedical systems allow for real-time triage and medical decision-making, ensuring that only critical cases are evacuated while non-emergency conditions are treated onboard under remote supervision. This targeted approach minimizes disruption to fishing operations and significantly reduces the financial strain on both maritime companies and national rescue services. Telemedicine also enables continuous health surveillance and preventive care, shifting the focus from reactive to proactive medical management. Wearable sensors and telemonitoring platforms collect physiological data such as heart rate, oxygen saturation, and stress indicators, which can be analyzed remotely to detect early signs of fatigue or illness. This continuous observation allows medical teams to provide early interventions, schedule rest periods, and recommend preventive measures before conditions escalate into emergencies. Such ongoing monitoring not only protects individual health but also contributes to maintaining overall operational efficiency. In addition, telemedicine enhances occupational safety by integrating health management into daily maritime operations. The ability to monitor crew health in real time ensures that potential risks—whether related to physical fatigue, dehydration, or psychological stress—are identified promptly. The inclusion of training modules and digital guidance within telemedical systems also empowers crew members with essential first-aid and emergency management skills. Together, these elements create a safer working environment, promoting both immediate well-being and long-term sustainability for those engaged in pelagic fishing operations [69].

### 6.2. Challenges

Despite its transformative potential, telemedicine in maritime applications faces several persistent challenges that must be addressed for widespread and effective adoption. The foremost technical limitation lies in bandwidth capacity and connectivity reliability. Satellite communication remains the primary link between ship and shore, but it is often constrained by weather interference, limited data throughput, and high operational costs. These factors can affect the quality of real-time video consultations and delay data transmission, particularly during adverse sea conditions. While emerging 5G maritime networks and low-earth-orbit satellite systems promise improvement, current infrastructure still poses a significant bottleneck for seamless medical communication. Another critical challenge involves the training requirements for both crew members and medical officers. Since telemedicine depends heavily on accurate data collection and the proper use of diagnostic devices, insufficient training can compromise the reliability of transmitted medical information. Crew personnel must be familiar with using wearable monitors, portable ultrasound devices, and telecommunication systems to ensure meaningful interactions with onshore physicians. Likewise, medical officers require specialized training in remote consultation protocols and cross-platform data interpretation to optimize clinical outcomes. Data privacy, cybersecurity, and ethical considerations also present growing concerns. The transmission of sensitive medical information through cloud-based systems introduces potential vulnerabilities to unauthorized access, data breaches, or misuse. Ensuring compliance with global data protection standards—such as GDPR (General Data Protection Regulation) and maritime health regulations—is essential for safeguarding both personal privacy and institutional accountability. Encryption, anonymization, and secure authentication systems must be rigorously maintained to uphold ethical standards in digital healthcare delivery. Finally, telemedicine must be effectively integrated with existing maritime regulations and healthcare frameworks. The maritime sector operates under diverse international jurisdictions, each with distinct health, safety, and legal requirements. Achieving regulatory harmonization across these systems remains a challenge, as does defining liability in cases of remote medical misjudgment. Collaboration between maritime authorities, healthcare providers, and telecommunication agencies is necessary to develop standardized protocols that ensure interoperability, legal clarity, and consistent quality of care across fleets and regions [57].

## 7. Future Perspectives

The future of maritime telemedicine lies in integrating high-speed communication, intelligent analytics, and interconnected health systems to provide continuous medical support in remote oceanic environments. The deployment of 5G-enabled maritime networks will overcome current bandwidth limitations, allowing for high-resolution video consultations, real-time data streaming from wearable devices, and faster diagnostic communication between vessels and onshore medical centers. Combined with edge computing and low-earth-orbit satellite systems, 5G will create a stable and resilient framework for uninterrupted healthcare delivery at sea. The IoMT will further transform vessel-based healthcare by linking wearable biosensors, portable diagnostics, and cloud platforms into a unified digital ecosystem. Through continuous data exchange, IoMT can monitor vital signs, predict fatigue or dehydration, and enable proactive interventions to prevent medical emergencies among fishermen working in physically demanding pelagic operations. Integration of AI-enhanced telehealth systems will enable predictive diagnostics and personalized health management. Commonly used AI frameworks in remote and maritime telehealth include LSTM models for predicting physiological time-series data such as heart rate variation and fatigue progression, CNNs for image-based evaluation of wounds or burns transmitted from vessels, and Bayesian models for recognizing stress-related behavioral patterns during prolonged offshore work. These approaches support early identification of health risks and provide decision support in settings where skilled medical personnel are unavailable. By analyzing longitudinal physiological and behavioral data, AI can identify early disease markers, forecast fatigue or stress, and recommend corrective actions. These systems will extend telemedicine beyond emergency care to include chronic disease monitoring and mental health assessment, providing holistic well-being support for seafarers exposed to isolation and long working hours. To ensure global scalability, standardized policy and regulatory frameworks are needed to address data security, interoperability, and ethical governance. Collaborative efforts between international maritime and health organizations will be essential for harmonizing telemedical standards and ensuring equitable access to digital healthcare technologies worldwide. In essence, the convergence of 5G, IoMT, and AI will redefine maritime telemedicine as a predictive, connected, and globally regulated healthcare system—ensuring continuous, high-quality medical care for fishermen in pelagic fisheries [70].

## 8. Conclusions

Telemedicine has revolutionized healthcare accessibility for fishermen operating in pelagic fisheries by bridging the vast physical divide between sea and shore. Through satellite-based communication, wearable health monitoring devices, and cloud-integrated data systems, medical professionals can now provide real-time diagnosis, guidance, and preventive care to crews working in the most remote maritime environments. This transformation ensures that medical assistance, once limited by distance and time, is now immediate, continuous, and data-driven. The integration of wearable technologies, satellite connectivity, and artificial intelligence analytics has created a synergistic framework that enables continuous health surveillance, predictive diagnostics, and personalized intervention. These technologies collectively enhance safety, reduce medical evacuation costs, and promote long-term well-being by turning vessels into mobile healthcare units capable of autonomous monitoring and communication. Looking ahead, sustained progress in maritime telemedicine will depend on international collaboration to establish standardized frameworks for interoperability, cybersecurity, and ethical governance. Global partnerships among healthcare institutions, maritime organizations, and telecommunication providers are essential to scale these innovations and ensure equitable, resilient telehealth coverage for all seafarers. By embracing such collaboration, telemedicine can evolve into a universal system that safeguards the health, productivity, and dignity of maritime workers across the world’s oceans. Although artificial intelligence-enabled telemedicine shows strong promise for maritime healthcare, further field-based validation studies conducted directly in pelagic fisheries are required to confirm performance, reliability, and operational feasibility under real fishing conditions.

## Figures and Tables

**Table 1:** Overview of Publications by Type and Environmental Context.

Type of Article	Study Environment	Categorized by Topic	Ref.
Pilot studies	Maritime	Wearable Digital Healthcare Devices	[18]
	Maritime	Digitally supported health management	[19]
	Maritime	Ultrasound	[20]
	Jungle	Feasibility of equipment & Video conferencing	[21]
	Mountain	Ultrasound	[22]
	Multiple austere environments	Ultrasound	[23]
	Antarctic	Feasibility of equipment	[24]
	Multiple austere environments	Telementoring	[25]
	Mountain	Telemonitoring	[26]
Cases (Reports/Series)	Maritime	Photography	[27]
	Maritime	–	[28]
	Maritime	AI-powered telemedicine	[29]
	Maritime	Photography	[30]
	Antarctic	Ultrasound	[31]
	Maritime	Photography	[32]
	Antarctic	General use of telemedicine	[33]
	Maritime	Specialties	[34]
	Mountain	Feasibility of equipment	[35]
Qualitative studies	Maritime	General use of telemedicine	[36]
	Maritime	User satisfaction	[37]
	Maritime		[28]
Company reports	Maritime	Company reports	[38]
	Maritime	Company reports	[39]
	Antarctic	Company reports	[40]
	Antarctic	Company reports	[41]
Retrospective studies	Maritime	Telemonitoring	[42]
	Maritime	AED	[43]
	Maritime	ECG	[44]
	Multiple austere environments	Specialties	[45]
	Antarctic	Ultrasound	[46]
Commentaries	Maritime	–	[47]
	Maritime	–	[48]
	Maritime	General use of telemedicine	[49]

**Table 2:** Overview of Examined Studies: Study Type, Sample, Year, and Principal Observations.

Ref.	Study Type	Year	Sample	Medical Advice	Observations
[50]	Retrospective cohort	2019	1401	Healthcare guidance for seafarer injuries	Non-officers and European seafarers face higher injury risks on Danish-flagged merchant ships
[51]	Observational	2017	169	Helicopter emergency evacuations (helivacs) between the two ferries	One person was airlifted every two weeks, mostly for cardiac cases, exceeding ambulance transfers
[52]	Epidemiological	2021	423	Support for managing seafarer injuries and illnesses	Non-officers reported far more injuries and illnesses than officers
[53]	Descriptive	2016	551	Cardiac symptom management	Pre-employment medical exams enhanced preventive care.
[3]	Retrospective	2020	11,481	Implementing preventive measures for COVID-19 control	Fever, sore throat, and shortness of breath were more prevalent during coronavirus outbreaks.
[54]	Observational	2019	225	Guarantee continuous medical support for seafarers	Delivering medical care for seafarers’ diverse health issues requires close multidisciplinary collaboration among medical officers
[55]	Observational	2022	384	Diagnosis of COVID-19	Encourages social distancing and quarantine measures at sea to curb pandemic spread.
[32]	Case study	2017	5	Medical conditions were diagnosed and monitored	In developing telemedical technologies, participants consistently show interest in photo-based teleconsultations
[56]	Cross-sectional	2022	420	Diagnosis of seafarers’ skin conditions	Noted inadequate remote care for dermatological conditions.

**Table 3:** AI Applications in Healthcare: Evidence from Empirical Studies.

Types	Detection	Models	Reliability	Ref.
Machine learning	Fatigue	Decision Tree (DT)	82.6%	[59]
Machine learning	Stress	Random Forest (RF)	73%	[60]
Machine learning	Motions	*k*-nearest Neighborhood (*k*-NN)	78.4%	[61]
Machine learning	Stress	Naive Bayes (NB)	85.5%	[62]
Machine learning	Stress	SVM	80.3%	[63]
Deep Learning	Fatigue	BiLSTM	99.9%	[64]
Deep Learning	Fatigue	CNN	88.85%	[65]
Hybrid	Stress	RF + SVM	98%	[66]
Hybrid	Motions	CNN + LSTM + BiLSTM	98.38%	[67]
Hybrid	Stress	CNN + RNN	85.71%	[68]

## Data Availability

All data supporting the findings of this study are included in the article’s reference list and are publicly available.
